# Adolescent smoking: The relationship between cigarette consumption and BMI

**DOI:** 10.1016/j.abrep.2018.100153

**Published:** 2018-12-08

**Authors:** Molly Jacobs

**Affiliations:** College of Allied Health Science, Department of Health Sciences Information and Management, East Carolina University, 600 Moye Blvd., Mail Stop 668, Health Sciences Building 4340E, Greenville, NC 27834, United States of America

**Keywords:** Obesity, Adolescence, BMI

## Abstract

**Background:**

Studies relating cigarette smoking and body weight yield conflicting results. Weight-lowering effects in women and men have been associated with smoking, however, no effects on weight have been proven. This study examined the association between cigarette smoking and relative weight in adolescent males and females as they age into young adults.

**Methods:**

Data from the National Longitudinal Survey of Youth—a nationally representative survey conducted annually—was used for this analysis. The sample consists of 4225 males and females observed annually from 1997 at age 12 to 17 through 2011 at age 27 to 31. Hierarchical generalized models (HGM) assess the impact of smoking on the likelihood of having higher BMI controlling for demographic, household and environmental impacts. The second estimation considers the possibility that smoking is endogenous and utilizes a multinomial instrument (IV) for smoking level.

**Results:**

HGM models reveal a negative association between cigarette smoking and BMI for both males and females. Individuals who smoke more have lower BMI compared to infrequent or non-smokers. General health rating, region of residence and income were used instrument for smoking in a linear two-stage IV specification. The instrument is highly correlated with BMI and results mirror the HGM. Finally, models run on early, middle and advanced adolescents show that the relationship diminishes over time. The relationship between BMI and smoking decreases as females age but increases for males.

**Conclusions:**

Empirical models confirm an association cigarette consumption and BMI in both males and females. This negative relationship varies with age. It is important to identify health risks—obesity—and modifiable risk factors—smoking—that contribute to health disparities among adolescents. However, the increase in one risky behavior leading to the decrease in the prevalence of the other, complicates the issue. The higher prevalence of frequent cigarette uses among both adolescents and young adults of lower BMI suggest that smoking could be used curb or suppress appetite.

## Highlights

• The weight impact of tobacco use by adolescents, unlike adults, has not been conclusively determined.
• This study examines the relationship between cigarette use and the body weight (BMI) of high school aged youth.
• Since smoking can be considered endogeneous in weight studies, smoking is instrumented in a two-stage process.
• Cigarette use is associated with higher BMI, but magnitudes vary by age.
• Models run on early, middle and older adolescents show that the relationship diminishes over time.
• The relationship between BMI and smoking decreases as females age but increases for males.
• Overweight and obese adolescents were more frequent tobacco users.
• Results suggest that electronic and conventional tobacco has similar BMI associations when used independently.

## 1 Introduction

Most people who use tobacco begin during adolescence (Walker & Loprinzi, 2014). Over 4.7 million middle and high school students currently use tobacco (Singh, 2016). While adolescent tobacco use has declined substantially over the last 40 years, nearly one in 20 high school seniors smoke daily (Johnston et al., 2018). At the same time, obesity rates currently exceed 30% in most age groups (Flegal, Graubard, Williamson, & Gail, 2007; Mokdad et al., 1999). Overweight and obesity, especially in children and young adults, are now regarded as one of the main public health challenges (Cunningham, Kramer, & Narayan, 2014; Gortmaker & Taveras, 2014; WHO, 2004).

While previous research provides varied results regarding the relationship between body mass index (BMI) and smoking at various ages, this study provides a comprehensive analysis of adolescent smoking at three stages of youth development. It incorporates longitudinal, nationally representative data and incorporates two different statistical methods to assert the robustness of the relationship. This analysis not only examines the relationship between cigarette smoking and BMI controlling for age, region of residence and other confounding variables, but also to test whether this relationship changes as adolescents age into young adults. This paper proceeds with a brief discussion of these issues and the existing literature in Section 2. Section 3 outlines the data and analytical methods employed.

## 2 Background

While the negative health impacts benefits of smoking are unquestionable, the strong probability of subsequent weight gain has raised concerns about an unintended effect of anti-smoking policies on obesity rates. Chou, Grossman, and Saffer (2004) proport that this resulting weight gain is simply “the price that must be paid to achieve goals that are in general favored by society.” Indeed, the association between smoking and body weight has become a central issue in the obesity literature, but the accumulating evidence present conflicting results (Potter et al., 2004). Substantial racial, ethnic and regional differences exist in smoking rates. White teens are more likely to smoke than are black or Hispanics (Kann et al., 2016). Smoking is more typically in nonmetropolitan areas, and in the South and Midwest (Abuse, 2010).

Some, but not all, previous studies found that cigarette smokers weigh less than nonsmokers and former smokers are no heavier than nonsmokers (Flegal et al., 2009). Others find a direct link between smoking and substantial weight gain (Coates & Li, 1983; Froom, Mizoue et al., 1998; Froom et al., 1999; Hudmon, Gritz, Clayton, & Nisenbaum, 1999; Klesges, Meyers, Klesges, & LaVasque, 1989; Klesges et al., 1998; Klesges et al., 1997; Manley & Boland, 1983; Moffatt & Owens, 1991; Shimokata et al., 1989; Williamson et al., 1991). Others find that a substantial decrease in cigarette smoking has only a small effect on the prevalence of obesity (Eisenberg & Quinn, 2006; Flegal, Troiano, Pamuk, Kuczmarski, & Campbell, 1995). Fang, Ali, and Rizzo (2009) reveal a moderately negative relationship between cigarette smoking and BMI, but the negative relationship could be attributable to simultaneity and should be interpreted with caution (Chen, Yen, & Eastwood 1994).

Much of the trouble in previous analyses involves lack of an identification strategy or appropriate instrument for endogenous factors. The motivation of initiating and maintaining smoking among adolescent females is quite different than males (Fulkerson & French, 2003; Potter et al., 2004; Vidrine, Anderson, Pollak, & Wetter, 2006). Weight concerns among adolescent females—who are more concerned with weight than males—may be one such factor (Attie & Brooks-Gunn, 1987; George & Johnson, 2001). More females consider themselves overweight than males (Winter, de Guia, Ferrence, & Cohen, 2002) and believe that smoking helps control weight (George & Johnson, 2001; Klesges, Elliott, & Robinson, 1997) leading them to use smoking as a method of weight control (Camp, Klesges, & Relyea, 1993; French & Perry, 1996; French, Perry, Leon, & Fulkerson, 1994; Gerend, Boyle, Peterson, & Hatsukami, 1998; Klesges, Mizes, & Klesges, 1987).

Studies examining the relationship between BMI and smoking in adults, show that cigarette smokers had a lower BMI, on average, than non-smokers or never smokers (Jessen, Buemann, Toubro, Skovgaard, & Astrup, 2005; Sneve & Jorde, 2008). Nicotine has been found to have slight metabolic effects and suppress appetite (Kvaavik, Meyer, & Tverdal, 2004; Perkins, Epstein, Marks, Stiller, & Jacob, 1989). In longitudinal analyses, continuing smokers had a smaller increase in BMI than those who gave up smoking (Bush, Lovejoy, Deprey, & Carpenter, 2016; Perkins et al., 1989; Robertson, McGee, & Hancox, 2014). In those who quit smoking, there was a significant, positive relationship between number of cigarettes smoked and the subsequent increase in BMI. The impact of smoking on body weight could dissipate over time. Long-term smokers (20+ years) are heavier than never or former smokers, and heavy smokers are more likely to be obese than both other smokers and nonsmokers (Chiolero, Jacot-Sadowski, Faeh, Paccaud, & Cornuz, 2007; Clair et al., 2011).

While smoking is correlated with lower BMI for adults, this trend has not been observed in younger smokers (ages 16–24 years) (Mackay, Gray, & Pell, 2013). The weight control effects of smoking may not be consistent among individuals in their developmental years or in the initial stages of use. Smoking has a reported antiestrogenic effect in youth, which may reduce fat deposition leading to weight loss (Pauly & Slotkin, 2008; Windham, Mitchell, Anderson, & Lasley, 2005). One study finds a positive impact of smoking on youth BMI, but highlights gender differences with females being more likely to initiate smoking and sustaining weight effects thereafter (Young et al., 2015).

In additional to the impact on body weight, the motivation for adolescent smoking is also unclear (Dierker & Mermelstein, 2010; Harris, Gordon-Larsen, Chantala, & Udry, 2006). A variety of factors have been identified as possible explanatory factors in use of substances other than smoking (Anderson, Pollak, & Wetter, 2002; Tyas & Pederson, 1998). Expectancy or trepidation for future events is among the most reliable correlates of substance experimentation, use, abuse, and dependence (Tyas & Pederson, 1998). Identifying factors that may mediate or moderate the smoking behavior is crucial for guiding the development of enhanced tobacco-control interventions targeting adolescents.

Next, Section 4 summarizes the empirical results and, finally, Section 5 discusses the results and primary conclusions.

## 3 Materials and methods

### 3.1 BMI

The Centers for Disease Control and Prevention (CDC) recommends using BMI percentiles—designed to capture the weight status of adolescents upon reaching young adulthood—to classify the body weight of individuals under age 18 and simple BMI values to classify weight of adults. Since respondents are age 12 to 17 in the first panel year, and quickly age beyond 19, BMI or the corresponding categorical ranking was used to classify weight in this analysis.^1^ BMI is highly correlated with body fat and provides a good indication of body size and fatness for most individuals. BMI is conventionally used to classify individuals as underweight, healthy weight, overweight, or obese using a nationally accepted rubric developed by the CDC (Flegal et al., 2009). Among adults, BMI appears to be a satisfactory measure of body fat especially if comparing across race and ethnicity (Burhkauser, Cawley, & Schmeiser, 2009; Mei et al., 2002).

BMI is assessed using data from the National Longitudinal Survey of Youth 1997 (NLSY97)—a longitudinal panel that follows a sample of 8984 American youth from 1997 to 2011. After 2011, the survey became biennial. While 2013 and 2015 are publicly available, this study will focus only on those consecutive survey years.

BMI was calculated from self-reported height and weight. To maintain a balanced panel, the sample includes only respondents with a BMI value in each year of the panel. While measurement and misspecification error is a concern in self-reported data, the data was cleaned to remove errant, inconsistent, and illogical values of height and weight. If BMI values were missing due to omitted height, height was imputed from nearby observations wherever possible. Full height is achieved relatively early in the panel; thus, imputations were unlikely to cause bias the sample. Imputations were needed on <8% of observations and final sample size was 4205 individuals—all comprising complete records throughout the panel. BMI and other means are listed in Table 1. Minimum BMI minimum is 12.5—underweight—and maximum is 55—overweight or obese—with an average of 25 and 26 for men and women respectively. BMI increases with age due to biological growth and weight gain but rates vary by race and gender (Gallagher et al., 1996).

**Table 1:** Descriptive Statistics of Covariates.

NLSY97: Descriptive statistics by gender					
	Obs	Mean	Std dev	Min	Max
Male					
BMI	29,786	25.83	5.21	14.137	54.81
Age	29,786	22.76	4.55	11	32
Black	29,786	0.22	0.41	0	1
Hispanic	29,786	0.19	0.39	0	1
South	29,786	0.36	0.48	0	1
Northeast	29,786	0.16	0.37	0	1
Urban	29,786	0.75	0.44	0	1
Household size	29,783	3.51	1.67	1	19
Income/poverty	20,842	390.09	376.71	1	3227
General health score	29,780	1.98	0.91	1	5
Body perception	29,654	3.20	0.74	1	5
Days smoked in last 30	10,479	20.57	11.65	1	30
Smoking category	29,786	0.81	1.20	0	3
Weight category	29,786	1.65	0.80	0	3

Female					
BMI	27,830	24.86	5.72	12.53	54.87
Age	27,830	22.67	4.60	11	32
Black	27,830	0.25	0.43	0	1
Hispanic	27,830	0.19	0.39	0	1
South	27,830	0.38	0.49	0	1
Northeast	27,830	0.16	0.36	0	1
Urban	27,830	0.77	0.42	0	1
Household size	27,829	3.64	1.72	1	15
Income/poverty	19,597	354.29	359.84	1	3227
General health score	27,827	2.17	0.93	1	5
Body perception	27,776	3.48	0.78	1	5
Days smoked in last 30	8701	21.04	11.58	1	30
Smoking category	27,830	0.73	1.17	0	3
Weight category	27,830	1.48	0.83	0	3

**Fig. 1:**
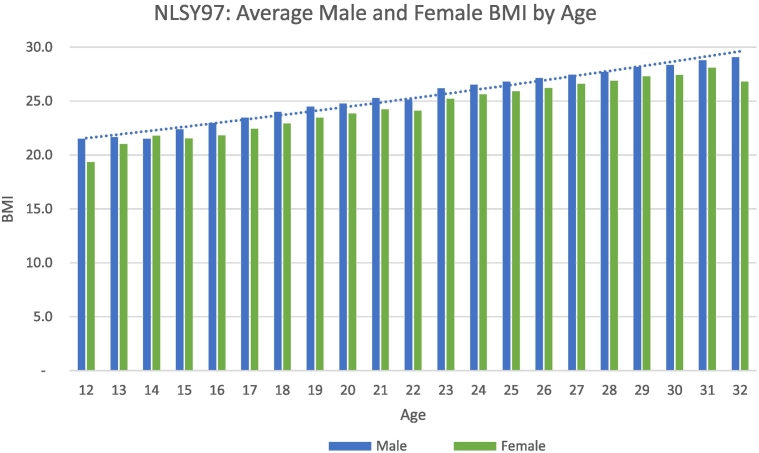
Average BMI by Age.

Analysis tests the relationship between BMI and cigarette smoking and is performed separately for men and women due to inherent biological differences and varying growth rates. BMI increases substantially over the panel with biological growth and increases in body fatness (Fig. 1). These data are consistent with other samples showing that BMI is comparatively higher among Hispanic males and black females. They also experience steeper growth trajectories (Kumar, Holmboe-Ottesen, Lien, & Wandel, 2004; Ogden, Stavrinaki, & Stubbs, 2009). The proportion of underweight decreased with age among all racial and ethnic groups and BMI levels remained high through adulthood.

### 3.2 Covariates

Average household size is 3.5 persons but decreases with age. Seventy-five percent of the sample resides in an urban area, compared to 80% of the US population (American Community Survey, 2017). Dummy variables, northeast and south, represent regional residence, and the income/poverty ratio accounts for income level. Ratios below 1 indicate an income below poverty, while ratios of one or greater indicate income at least at the poverty level. The average ratio in the sample is between five and six—above poverty level.

General health score classifies overall health as 1 = excellent, 2 = very good, 3 = good, 4 = fair or 5 = poor. The higher the rating, the lower the general level of health. On average, men and women rate their general health as 2 to 3 or “good”. While the survey includes many questions about drinking, smoking, sleep and exercise, much of the data is incomplete or only specified in a handful of panel years. To obtain a valid indicator of adolescent smoking, number of days smoked was chosen as the most completely, accurate measure. Response indicates whether they smoke zero or 1 to 5, 6 to 10, …, or 26 to 30 days a month. Most respondents indicate that they are non-smokers, smoking zero out of 30 days. Among those who report smoking, the average number of days smoked is between 20 and 21.

Smoking categories correspond to the American Heart Association labels of never-smokers or non-smokers, light smokers, moderate smokers and heavy smokers based on both average number of cigarettes and days smoked. Table 2 shows the average frequency and proportion of men in women in each weight and smoking category. Most respondents are normal weight or overweight and report being non-smokers. The greatest public health concern lies with the 18% of men and 16% of women who are both obese and smoke heavily.

**Table 2:** Categorical representation and criteria.

NLSY97: Categorical representation and criteria					
	N	Pct	N	Pct	Criteria
	Male		Female		
Smoking category					
Non-smoker	19,307	64.82	19,129	68.74	0 days smoking
Light smoker	2413	8.1	1940	6.97	0–5 days smoking
Moderate smoker	2633	8.84	2044	7.34	6–20 days smoking
Heavy smoker	5433	18.24	4717	16.95	21–30 days smoking

Weight category					
Underweight	787	2.64	1499	5.39	BMI ≤ 18.5
Normal weight	14,125	47.42	15,966	57.37	18.5 < BMI ≤ 24.9
Overweight	9485	31.84	5840	20.98	25 ≤ BMI < 30.0
Obese	5389	18.09	4525	16.26	30 ≤ BMI

Table 3 provides cross frequencies of smoking and weight categories. Most respondents in all weight categories are non-smokers. Between 15% of men and 20% of women are heavy smokers. These simple statistics suggest that there are more underweight and obese heavy smokers than other groups especially for women.

**Table 3:** Proportion of weight category by smoking frequency.

NLSY97: Proportion of weight category by smoking frequency				
Percent Row pct Col pct	Underweight	normal weight	Overweight	Obese
Male				
Nonsmoker	12.23	20.55	30.07	1.97
	18.87	31.7	46.39	3.04
	67.6	64.53	63.41	74.46
Light smoker	1.47	2.55	3.93	0.15
	18.11	31.5	48.57	1.82
	8.11	8.01	8.3	5.59
Moderate smoker	1.75	2.9	4.06	0.13
	19.75	32.85	45.88	1.52
	9.65	9.12	8.55	5.08
Heavy smoker	2.65	5.84	9.36	0.39
	14.52	32.01	51.32	2.15
	14.64	18.33	19.74	14.87

Female				
Nonsmoker	10.82	14.44	39.78	3.7
	15.74	21.01	57.87	5.38
	66.54	68.82	69.33	68.65
Light smoker	0.83	1.33	4.5	0.31
	11.96	19.07	64.48	4.48
	5.13	6.34	7.84	5.8
Moderate smoker	1.27	1.5	4.23	0.34
	17.27	20.45	57.63	4.65
	7.8	7.16	7.38	6.34
Heavy smoker	3.34	3.71	8.86	1.03
	19.69	21.9	52.3	6.11
	20.53	17.69	15.45	19.21

### 3.3 Statistical analysis

Data are analyzed using SAS 9.4 (Cary, NC). First, a hierarchical generalized model (HGM) was used. HGMs are appropriate only when the outcome of interest is not normally distributed, such as weight category, an appropriate error distribution needs to be incorporated into the model. Previously presented by Bell, Ene, Smiley and Schoeneberger (2013), HGM is a two-level organizational model with a polytomous outcome—the BMI category of youth drawn from a nationally representative longitudinal sample of American youth. HGMs accommodate categorical, non-normally distributed response variables. When dealing with this type of model, the assumptions of normally distributed, homoscedastic errors are violated (Hox et al., 2002; O'Connell, Goldstein, Rogers & Peng, 2008). Therefore, model employs a transformation of the BMI category using a cumulative logit link function and a multinomial distribution. These models are used to assess the relationship between hierarchical BMI category, smoking and demographic controls.

Research concerning the association between smoking and body weight lacks consistency and has several weaknesses (Shimokata, Muller, & Andres, 1989; Williamson et al., 1991; Yen, Chen, & Eastwood, 2009). For example, estimation of the impact of smoking on body weight, like all statistical models, could be biased unobserved personal characteristics that motivate smoking (French & Popovici, 2011; Leigh & Schembri, 2004).

The absence of a mechanism for modeling the endogeneity of smoking choices has challenged researchers has been confronted in various ways (Kasteridis & Yen, 2012). When the unobservable motivations for smoking are omitted variables correlated with included regressors, standard estimation methods will generally be inconsistent. Though alternative consistent estimators may exist in special circumstances, it is suggested here that a nonlinear instrumental-variable strategy offers a reasonably general solution to such estimation problems. A variety of different instruments have been used to control for smoking decision—Vietnam war draft (De Walque, 2007), infant neurodevelopment (Wehby, Prater, McCarthy, Castilla, & Murray, 2011) and schooling and earnings (Angrist & Imbens, 1995)—by utilizing a two-stage, maximum likelihood estimation. While the instruments vary in their exact specification, the incorporate common elements of regional, social, economic and health measures. The instrument in this study follows the same logic. Smoking behavior is instrumented using region, income, age and general health status. The logic of the instrumentation equation is simple—higher smoking rates in the south, high cost of cigarettes and related taxes, and the negative health impacts of cigarette use.

## 4 Results

Table 4 lists estimation results from the HGM specification. Since underweight is the reference category, estimates model the probability of having a lower BMI category. The negative age coefficient indicates that as age increases (Odds Ratio (OR) = 1.44, 95% Confidence Interval (CI) = 1.398–1.468) respondents have a lower likelihood of having a low BMI category. In other words, BMI increases with age. Most regional and geographic coefficients are insignificant. Smoking is insignificant for females suggesting that as smoking frequency increases, so does the probability of having a lower BMI category.

**Table 4:** HGLM results.

NLSY97: HLGM results by gender					
Male			Female		
Value	BMI category	Observations	Value	Smoking category	Observations
0	Underweight	115	0	Underweight	278
1	Normal Weight	3605	1	Normal Weight	3353
2	Overweight	2602	2	Overweight	1419
3	Obese	1370	3	Obese	1235

Modeling the probability of having a lower BMI category					
Fit statistics			Fit statistics		
−2 log likelihood	10,011.78		−2 log likelihood	8600.51	
AIC	10,038		AIC	8626.51	

Results			Results		
Effect	Estimate	Std dev	Effect	Estimate	Std dev
Intercept (normal)	−1.8481***	0.4531	Intercept (normal)	0.3189	0.4367
Intercept (over)	8.1182***	0.4526	Intercept (over)	9.7646***	0.4825
Intercept (obese)	12.1022***	0.4905	Intercept (obese)	13.2557***	0.5149
Smoking	0.004543	0.00459	Smoking	0.008554*	0.005218
Age	−0.3586***	0.01255	Age	−0.3188***	0.01283
South	−0.042	0.2053	South	−0.1393	0.2116
Northeast	0.2858	0.3008	Northeast	0.2843	0.2865
Black	0.1025	0.3683	Black	−3.14***	0.4171
Hispanic	−1.3365***	0.3669	Hispanic	−0.8869**	0.408
Urban	0.1091	0.1122	Urban	−0.1711	0.1158
Household size	0.01609	0.02822	Household size	−0.08449**	0.03001
logIncome/poverty	0.03824	0.04167	logIncome/poverty	0.0382**	0.04456

Dependent variable: BMI category.

1 = underweight, 2 = normal weight, 3 = overweight, 4 = obese.

Dependent variable: BMI category.

1 = underweight, 2 = normal weight, 3 = overweight, 4 = obese.

* = 10%, ** = 5%, *** = 1%.

Racial and ethnic variables appear highly deterministic. Hispanic males are significantly less likely than white males to be low BMI (OR = 5.201, 95% CI = 2.527–10.705) while black women are less likely to be low BMI, all else held constant (OR = 23.118, 95% CI = 10.207–52.360). This is consistent with other studies who found that black women and Hispanic men are heavier due, in part, to body size preference (Cachelin, Rebeck, Chung, & Pelayo, 2002). Demographic and socioeconomic factors, such as race, ethnicity and income, contribute significantly to health disparities among adolescents and young adults (Eberhardt et al., 2001; Pamuk, Makuc, Heck, Reuben, & Lochner, 1998). For women, household size and income are negatively and positively, respectively, related to BMI category, but not among men. Adolescent women in large households are less likely to be low BMI (OR = 1.088, 95% CI = 1.026–1.154) and those with a higher income are more likely (OR = 0.962, 95% CI = 0.882–1.050).

Results from the two-stage regression are given in Table 5. The first stage regresses age, region of residence, race/ethnicity, household size, and income-poverty ratio on smoking frequency. Results show all covariates significant for females and most for males (F Statistic (F) = 62.27, p-value (p) < 0.0001). Smoking frequencies increase with age (15–20% per year, Standard Error (SE) = 0.038), southern residence and general health. An increase in smoking—a probable cause of poor health—corresponds to a poorer health rating (recall that higher numeric indicates lower health score), all else held constant. Income has an inverse relationship with smoking frequency indicating the smoking rates decline as income increases. The residuals from Stage 1 are retained and used to approximate smoking frequency in Stage 2. The Stage 2 regression model is run as a categorical dependent variable model with the created instrument. The instrumented value appears to be a valid instrument and is highly correlated with BMI category (SE = 0.00197, p = 0.0001).

**Table 5:** 2SLS Results.

NLSY97: 2SLS results by gender							
Male				Female			
Stage 1: Analysis of variance				Stage 1: Analysis of variance			
Source	Sum of squares	Mean square	F value	Source	Sum of squares	Mean square	F value
Model	32,292	8072.9387	62.97⁎⁎⁎	Model	36,333	9083.3424	74.41⁎⁎⁎
Error	985,309	128.21202		Error	766,511	122.07538	
Corrected total	1,017,601			Corrected total	802,845		
Stage 1: Model fit				Stage 1: Model fit			
Root MSE	11.32307	R-square	0.0317	Root MSE	11.04877	R-square	0.0453
Dependent mean	20.90468	Adj R-sq	0.0312	Dependent mean	21.65468	Adj R-sq	0.0446
Coeff var	54.16526			Coeff var	51.02257		
Stage 1: Parameter estimates				Stage 1: Parameter estimates			
Variable	Parameter	Standard	t value	Variable	Parameter	Standard	t value
Intercept	13.59121⁎⁎⁎	1.12	12.09	Intercept	14.788⁎⁎⁎	1.17929	12.54
Age	0.15279⁎⁎⁎	0.04	4.12	Age	0.20625⁎⁎⁎	0.03884	5.31
South	2.01406⁎⁎⁎	0.14	14.28	South	2.18845⁎⁎⁎	0.15085	14.51
General health rating	0.75664⁎⁎⁎	0.27	2.82	General health rating	0.57644⁎⁎	0.28974	1.99
logIncome/poverty	−0.19443	0.12	−1.57	logIncome/poverty	−0.65774⁎⁎⁎	0.12707	−5.18
Dependent variable: Smoking				Dependent variable: Smoking			

Stage 2: Response category				Stage 2: Response category			
Category	Range	Frequency		Category	Range	Frequency	
Underweight	≤18.5	115		Underweight	≤18.5	278	
Normal weight	18.5 < BMI < 25	3604		Normal weight	18.5 < BMI < 25	3353	
Overweight	25 ≤ BMI < 30	2602		Overweight	25 ≤ BMI < 30	1418	
Obese	≥30.0	1369		Obese	≥30.0	1235	
Stage 2: Model fit				Stage 2: Model fit			
Criterion	Intercept only	Intercept and covariates		Criterion	Intercept only	Intercept and covariates	
AIC	16,800.02	16,271.4		AIC	14,192.721	13,627.4	
SC	16,820.863	16,820.9		SC	14,212.959	13,708.4	
−2 log L	16,794.020	16,247.4		−2 log L	14,186.721	13,603.4	
Stage 2: Parameter estimates				Stage 2: Parameter estimates			
Variable	Parameter	Standard	Wald Chi square	Variable	Parameter	Standard	Wald Chi square
Intercept_0	−0.6781⁎⁎⁎	0.2238	9.1798	Intercept_0	−0.2549	0.2211	1.3292
Intercept_1	3.5676⁎⁎⁎	0.211	286.0026	Intercept_1	3.2908⁎⁎⁎	0.221	221.8288
Intercept_2	5.2498⁎⁎⁎	0.2157	592.3094	Intercept_2	4.4713⁎⁎⁎	0.2243	397.4238
Smoking	0.0127⁎⁎⁎	0.00197	41.1761	Smoking	0.00408⁎	0.00226	3.2599
Age	−0.1223⁎⁎⁎	0.00645	359.4032	Age	−0.1123⁎⁎⁎	0.00696	260.5239
Black	0.0538	0.05	1.1561	Black	0.00575	0.0552	0.0109
Hispanic	−0.0339	0.0633	0.286	Hispanic	0.0997	0.0698	2.0381
Urban	−0.1945⁎⁎⁎	0.0618	9.9102	Urban	−1.0698⁎⁎⁎	0.0701	232.901
South	−0.4853⁎⁎⁎	0.0623	60.7172	South	−0.3454⁎⁎⁎	0.0714	23.4023
Northeast	0.000333	0.0513	0	Northeast	−0.0118	0.0582	0.0407
Household size	−0.017	0.0138	1.5104	Household size	−0.0575⁎⁎⁎	0.0151	14.5525
logIncome/poverty	−0.1⁎⁎⁎	0.0216	21.3912	logIncome/poverty	0.0241	0.0229	1.1074
Dependent variable: BMI category				Dependent variable: BMI category			

⁎ Significance: 10%.

⁎⁎ Significance: 15%.

⁎⁎⁎ Significance: 1%.

Consistency of results reinforces the strength of the relationship between smoking and body weight and suggests that any endogeneity bias is not a substantially problem. Smoking frequency is negatively related to weight, but age and race/ethnicity are positively related (black for females and Hispanic for males). Minority groups have a lower probability of being in a low weight category—a sensible result given that they tend to have high average BMI. As expected, the probability of low BMI decreases with age for men and women but increases with household size among women. Men have a negative correlation between weight and income.

There appears to be a relationship between BMI and smoking, but does it vary with age? Young adults and adolescents might behave or respond differently to external stimuli. To test the robustness of the HGM and 2SLS models to age and BMI changes, models were run separately at three different points in the age distribution—age 12 to 17, 20 to 25 and 27 to 32. Results are listed in Appendix I. As males age, smoking increases in significance become more deterministic. For females, the opposite occurs—there is a strong relationship between BMI category and smoking for those age 12 to 17, but it decreases with age.

## 5 Discussion and conclusion

This paper addresses the following research areas:

1. How prevalent is overweight among males and females during the adolescent years?
2. Does this prevalence vary across demographic/household/geographic characteristics?
3. What is the relationship between smoking frequency and BMI?
4. Does the relationship between smoking and BMI change between adolescence and young adulthood?

Analysis showed that males and females gain weight with age and obesity/overweight become more prevalent over time. Smoking rates remain low but persist steadily throughout adolescence. Household and geographic patterns pay little role in BMI determination. Race, age and ethnicity are highly deterministic and positive—older and minority respondents have comparatively higher BMI. Household size plays a small role for females and income for males.

Finally, smoking and BMI are inversely related—lower BMI respondents smoke more. Higher BMI respondents tend to be light or non-smokers. When similar analysis was conducted in young, middle and older adolescents, males showed that the relationship between BMI and smoking frequency became stronger over time while women showed that smoking frequency became less deterministic. Causality falls outside the scope of the analysis, but reports show significantly higher smoking rates among men, but faster BMI increases among women. Therefore, both female smokers and non-smokers are likely to be increasing BMI more rapidly and the differential between the two groups could narrow. The disparity between male smokers and non-smokers could be growing as more males continue to smoke later in life or are unsuccessful quitters. This analysis shows a significant behavioral impact on BM, but the age-related relationship for men and women merits further analysis.

While analysis utilized statistical controls to obtain robust estimates, this study faces several limitations that deserve consideration. First, BMI calculations are based on self-reported height and weight which is subject to reporting bias. The clinical limitations of BMI should also be considered. BMI is a surrogate measure of body fatness because it is a measure of excess weight rather than excess body fat. Factors such as age, sex, ethnicity, and muscle mass can influence the relationship between BMI and body fat. Furthermore, this study does not consider the behavioral mechanisms underlying smoking or excessive body weight. As a result, these behaviors could manifest singularly or jointly in individuals based on underlying motivations.

Despite efforts, physicians and policy makers have not succeeded in reversing the trend of adolescent smoking or obesity (Roberto et al., 2015; Swinburn et al., 2011). Research indicates that public perception of overweight and obesity has been influenced, but public disfavor for smoking persists (Johnson, Cooke, Croker, & Wardle, 2008). Given the results of this study, public health campaigns should be tailored specifically for age groups experiencing similar phenomenon. Additionally, adolescents should be taught the difference between simple behavioral modification and behavioral replication so that the trade-off between cessation and weight gain does not become a societal norm.

The following is the supplementary data related to this article.

Appendix I Additional regression results. Appendix I

## Funding disclosure

The author reports no monetary interests in the publication of this manuscript. No external funding was used in the research contained herein.

## Ethical approval disclosure

This manuscript does not contain any studies with human participants or animals performed by the author.

## Conflicts of interest disclosure

The author certifies that he/she has NO affiliations with or involvement in any organization or entity with any financial interest (such as honoraria; educational grants; participation in speakers' bureaus; membership, employment, consultancies, stock ownership, or other equity interest; and expert testimony or patent-licensing arrangements), or non-financial interest (such as personal or professional relationships, affiliations, knowledge or beliefs) in the subject matter or materials discussed in this manuscript.

## Significance for public health research

While the weight lowering effects if cigarette smoking among adults is well documented, results among adolescents have been mixed. Previous studies have been plagued by the endogeneity of smoking in weight growth studies. This study over comes these difficulties by utilizing both standard hierarchical generalized regression and instrumental variables. Both models confirm the negative relationship between BMI and smoking frequency but show that the strength of the relationship varies as adolescents age. The strong age variation explains the mixed results found in earlier works. Understanding the cause behind adolescent weight disparities has important public health implications for designing and tailoring intervention programs.

## Footnotes

1 Using BMI, BMI percentiles and a hybrid of the two systems was tried. However, the hybrid system created a large discontinuity and percentiles are largely inapplicable to most of the sample. Therefore, only BMI was used.
