# Supplementary material for: Adolescent smoking: The relationship between cigarette consumption and BMI
Source: Addict Behav Rep. 2018 Dec 8;9:100153. doi: 10.1016/j.abrep.2018.100153 (PMC6542372; doi:10.1016/j.abrep.2018.100153)
Supplement: Appendix I — Additional regression results. [file mmc1.docx]

Appendix I

| NLSY97: 2SLS Results by Gender | | | | | | | |
| --- | --- | --- | --- | --- | --- | --- | --- |
| Male | | | | Female | | | |
| Age 12-19 | | | | | | | |
| Stage 1: Analysis of Variance | | | | Stage 1: Analysis of Variance | | | |
| Source | Sum of Squares | Mean Square | F Value | Source | Sum of Squares | Mean Square | F Value |
| Model | 6701.01244 | 1675.25311 | 11.99*** | Model | 6970.41521 | 1742.6038 | 12.85*** |
| Error | 105933 | 139.75311 |  | Error | 95600 | 135.60338 |  |
| Corrected Total | 112634 |  |  | Corrected Total | 102571 |  |  |
| Stage 1: Model Fit | | | | Stage 1: Model Fit | | | |
| Root MSE | 11.82172 | R-Square | 0.0595 | Root MSE | 11.64489 | R-Square | 0.068 |
| Dependent Mean | 19.29882 | Adj R-Sq | 0.0545 | Dependent Mean | 19.73944 | Adj R-Sq | 0.0627 |
| Coeff Var | 61.25619 |  |  | Coeff Var | 58.993 |  |  |
| Stage 1: Parameter Estimates | | | | Stage 1: Parameter Estimates | | | |
| Variable | Parameter | Standard | t Value | Variable | Parameter | Standard | t Value |
| Intercept | -4.11264 | 5.42697 | -0.76 | Intercept | 0.22196 | 5.58302 | 0.04 |
| Age | 1.14408*** | 0.27395 | 4.18 | Age | 1.17099*** | 0.28284 | 4.14 |
| General Health Rating | 2.4515*** | 0.46274 | 5.3 | General Health Rating | 2.19796*** | 0.47966 | 4.58 |
| South | 0.34278 | 0.88589 | 0.39 | South | 0.39314 | 0.9142 | 0.43 |
| Income/Poverty | -0.45992 | 0.39082 | -1.18 | Income/Poverty | -1.33182*** | 0.37591 | -3.54 |
| Dependent Variable: Smoking | | | | Dependent Variable: Smoking | | | |
| Stage 2: Analysis of Variance | | | | Stage 2: Analysis of Variance | | | |
| Criteria | Intercept Only | Intercept and Covariates | | Criteria | Intercept Only | Intercept and Covariates | |
| AIC | 1429.988 | 1427.496 | | AIC | 1466.29 | 1438.99 | |
| SC | 1443.9 | 1483.144 | | SC | 1479.99 | 1493.77 | |
| -2 Log L | 1423.988 | 1403.496 | | -2 Log L | 1460.29 | 1414.99 | |
| Stage 2: Model Fit | | | | Stage 2: Model Fit | | | |
| Likelihood Ratio | 20.492 |  |  | Likelihood Ratio | 45.3044 |  |  |
| Score | 20.3582 |  |  | Score | 43.7817 |  |  |
| Wald | 20.0964 |  |  | Wald | 46.6492 |  |  |
| Stage 2: Parameter Estimates | | | | Stage 2: Parameter Estimates | | | |
| Variable | Parameter | Standard | Wald Chi Square | Variable | Parameter | Standard | Wald Chi Square |
| Intercept | -0.9755 | 1.0214 | 0.912 | Intercept | -0.4342 | 1.0221 | 0.1805 |
| Intercept | 3.1801*** | 1.0242 | 9.6414 | Intercept | 3.0515*** | 1.0318 | 8.7472 |
| Intercept | 4.8716*** | 1.0335 | 22.2205 | Intercept | 4.3603 | 1.0383 | 17.6355 |
| Smoking | 0.0106 | 0.00648 | 2.6739 | Smoking | 0.00156*** | 0.00677 | 0.0529 |
| Age | -0.1154 | 0.0515 | 5.0315 | Age | -0.1376** | 0.0517 | 7.09 |
| South | -0.0683 | 0.1714 | 0.1588 | South | 0.1286*** | 0.1723 | 0.5567 |
| Northeast | -0.42441** | 0.2172 | 3.8192 | Northeast | 0.2432 | 0.2248 | 1.1704 |
| Black | -0.3126 | 0.2182 | 2.0515 | Black | -1.0543*** | 0.2277 | 21.4345 |
| Hispanic | -0.1595 | 0.2202 | 0.5248 | Hispanic | -0.3126 | 0.226 | 1.912 |
| Urban | 0.0629 | 0.1709 | 0.1352 | Urban | -0.0281 | 0.1793 | 0.0245 |
| Household Size | -0.0926* | 0.0503 | 3.3854 | Household Size | -0.0678 | 0.0504 | 1.8105 |
| logIncome/Poverty | 0.0462 | 0.0712 | 0.4207 | logIncome/Poverty | 0.1668** | 0.0672 | 6.1625 |
| Age 20-25 | | | | | | | |
| Stage 1: Analysis of Variance | | | | Stage 1: Analysis of Variance | | | |
| Source | Sum of Squares | Mean Square | F Value | Source | Sum of Squares | Mean Square | F Value |
| Model | 11589 | 2897.35074 | 22.73*** | Model | 15297 | 3824.35525 | 31.41*** |
| Error | 453271 | 127.46666 |  | Error | 348466 | 121.75617 |  |
| Corrected Total | 464861 |  |  | Corrected Total | 363764 |  |  |
| Stage 1: Model Fit | | | | Stage 1: Model Fit | | | |
| Root MSE | 11.29011 | R-Square | 0.0249 | Root MSE | 11.03432 | R-Square | 0.0421 |
| Dependent Mean | 20.88992 | Adj R-Sq | 0.0238 | Dependent Mean | 21.57272 | Adj R-Sq | 0.0407 |
| Coeff Var | 54.04575 |  |  | Coeff Var | 51.1494 |  |  |
| Stage 1: Parameter Estimates | | | | Stage 1: Parameter Estimates | | | |
| Variable | Parameter | Standard | t Value | Variable | Parameter | Standard | t Value |
| Intercept | 13.78469*** | 3.25427 | 4.24 | Intercept | 18.32528*** | 3.52324 | 5.2 |
| Age | 0.08364 | 0.13406 | 0.62 | Age | 0.00115*** | 0.14814 | 0.01 |
| General Health Rating | 1.89904*** | 0.20659 | 9.19 | General Health Rating | 2.37701*** | 0.22432 | 10.6 |
| South | 1.01242 | 0.39262 | 2.58 | South | 0.64716 | 0.42985 | 1.51 |
| Income/Poverty | 0.13535 | 0.1766 | 0.77 | Income/Poverty | -0.49097* | 0.18703 | -2.63 |
| Dependent Variable: Smoking | | | | Dependent Variable: Smoking | | | |
| Stage 2: Analysis of Variance | | | | Stage 2: Analysis of Variance | | | |
| Criteria | Intercept Only | Intercept and Covariates | | Criteria | Intercept Only | Intercept and Covariates | |
| AIC | 7689.381 | 7596.58 | | AIC | 6363.944 | 6199.67 | |
| SC | 7707.915 | 7670.71 | | SC | 6381.827 | 6271.2 | |
| -2 Log L | 7683.381 | 7572.58 | | -2 Log L | 6357.944 | 6175.67 | |
| Stage 2: Model Fit | | | | Stage 2: Model Fit | | | |
| Likelihood Ratio | 110.8067 |  |  | Likelihood Ratio | 182.2781 |  |  |
| Score | 110.5945 |  |  | Score | 180.1403 |  |  |
| Wald | 108.5867 |  |  | Wald | 179.403 |  |  |
| Stage 2: Parameter Estimates | | | | Stage 2: Parameter Estimates | | | |
| Variable | Parameter | Standard | Wald Chi Square | Variable | Parameter | Standard | Wald Chi Square |
| Intercept | -0.9057 | 0.5735 | 2.494 | Intercept | 0.4249 | 0.6315 | 0.4526 |
| Intercept | 3.3848*** | 0.5613 | 36.3681 | Intercept | 4.014*** | 0.6327 | 40.2526 |
| Intercept | 5.0225*** | 0.5649 | 79.0593 | Intercept | 5.2118*** | 0.6357 | 67.2096 |
| Smoking | 0.012*** | 0.00289 | 17.199 | Smoking | -0.00088 | 0.00336 | 0.0681 |
| Age | -0.123*** | 0.0228 | 29.0796 | Age | -0.1361*** | 0.0262 | 26.9597 |
| South | -0.0227 | 0.073 | 0.0971 | South | -0.00214 | 0.0818 | 0.0007 |
| Northeast | -0.0377 | 0.0938 | 0.162 | Northeast | 0.067 | 0.1038 | 0.4165 |
| Black | -0.0845 | 0.0924 | 0.8365 | Black | -1.1518*** | 0.1051 | 120.1069 |
| Hispanic | -0.5678*** | 0.0912 | 38.7375 | Hispanic | -0.4163*** | 0.1028 | 16.3853 |
| Urban | 0.087 | 0.0756 | 1.3245 | Urban | 0.0409 | 0.0877 | 0.2171 |
| Household Size | 0.00795 | 0.0207 | 0.1481 | Household Size | -0.0871 | 0.0226 | 14.8811 |
| logIncome/Poverty | -0.0984** | 0.0306 | 10.3402 | logIncome/Poverty | -0.00461 | 0.0336 | 0.0188 |
| Age 27-32 | | | | | | | |
| Stage 1: Analysis of Variance | | | | Stage 1: Analysis of Variance | | | |
| Source | Sum of Squares | Mean Square | F Value | Source | Sum of Squares | Mean Square | F Value |
| Model | 11155 | 2788.79264 | 22.58*** | Model | 9071.01327 | 2267.75332 | 19.43*** |
| Error | 335160 | 123.49295 |  | Error | 254024 | 116.68547 |  |
| Corrected Total | 346315 |  |  | Corrected Total | 263095 |  |  |
| Stage 1: Model Fit | | | | Stage 1: Model Fit | | | |
| Root MSE | 11.11274 | R-Square | 0.0322 | Root MSE | 10.8021 | R-Square | 0.0345 |
| Dependent Mean | 21.59139 | Adj R-Sq | 0.0308 | Dependent Mean | 22.51008 | Adj R-Sq | 0.0327 |
| Coeff Var | 51.46837 |  |  | Coeff Var | 47.98785 |  |  |
| Stage 1: Parameter Estimates | | | | Stage 1: Parameter Estimates | | | |
| Variable | Parameter | Standard | t Value | Variable | Parameter | Standard | t Value |
| Intercept | 19.21129*** | 4.02301 | 4.78 | Intercept | 19.5725*** | 4.28421 | 4.57 |
| Age | 0.06907 | 0.13923 | 0.5 | Age | 0.082 | 0.1449 | 0.57 |
| General Health Rating | 1.87715*** | 0.23394 | 8.02 | General Health Rating | 1.79565*** | 0.2474 | 7.26 |
| South | 0.98964** | 0.44514 | 2.22 | South | 0.60026 | 0.47785 | 1.26 |
| Income/Poverty | -0.78165*** | 0.21961 | -3.56 | Income/Poverty | -0.74691*** | 0.22245 | -3.36 |
| Dependent Variable: Smoking | | | | Dependent Variable: Smoking | | | |
| Stage 2: Analysis of Variance | | | | Stage 2: Analysis of Variance | | | |
| Criteria | Intercept Only | Intercept and Covariates | | Criteria | Intercept Only | Intercept and Covariates | |
| AIC | 6055.676 | 5974.97 | | AIC | 5104.473 | 5008.97 | |
| SC | 6073.4 | 6045.86 | | SC | 5121.537 | 5077.23 | |
| -2 Log L | 6049.676 | 5950.97 | | -2 Log L | 5098.473 | 4984.97 | |
| Stage 2: Model Fit | | | | Stage 2: Model Fit | | | |
| Likelihood Ratio | 98.7099 |  |  | Likelihood Ratio | 113.501 |  |  |
| Score | 98.8818 |  |  | Score | 112.4363 |  |  |
| Wald | 95.2413 |  |  | Wald | 109.8665 |  |  |
| Stage 2: Parameter Estimates | | | | Stage 2: Parameter Estimates | | | |
| Variable | Parameter | Standard | Wald Chi Square | Variable | Parameter | Standard | Wald Chi Square |
| Intercept | -1.3794* | 0.718 | 3.6908 | Intercept | -1.1894 | 0.7581 | 2.4619 |
| Intercept | 3.0008*** | 0.6886 | 18.9915 | Intercept | 2.2406** | 0.7524 | 8.8681 |
| Intercept | 4.7536*** | 0.6926 | 47.1112 | Intercept | 3.367*** | 0.7544 | 19.9205 |
| Smoking | 0.0145*** | 0.00335 | 18.7311 | Smoking | 0.00699 | 0.00382 | 3.3546 |
| Age | -0.0746** | 0.0233 | 10.2859 | Age | -0.0777** | 0.0251 | 9.5861 |
| South | 0.1234 | 0.083 | 2.2129 | South | -0.0243 | 0.0909 | 0.0715 |
| Northeast | 0.0378 | 0.1043 | 0.1312 | Northeast | 0.089 | 0.114 | 0.6102 |
| Black | -0.2985*** | 0.0985 | 9.1912 | Black | -0.9073*** | 0.1121 | 65.4798 |
| Hispanic | -0.442*** | 0.1025 | 18.5803 | Hispanic | -0.2425** | 0.1214 | 3.9908 |
| Urban | -0.1351 | 0.085 | 2.5252 | Urban | -0.0919 | 0.0961 | 0.9139 |
| Household Size | -0.0557** | 0.023 | 5.8399 | Household Size | -0.0454* | 0.0253 | 3.2248 |
| logIncome/Poverty | -0.1995*** | 0.0394 | 25.5905 | logIncome/Poverty | 0.0462 | 0.041 | 1.2686 |
| Dependent Variable: BMI Category | | | | Dependent Variable: BMI Category | | | |
| Significance: * = 10%, ** = 15%, *** = 1% | | | | Significance: * = 10%, ** = 15%, *** = 1% | | | |
| Modeling the probability of having a lower BMI Category | | | | Modeling the probability of having a lower BMI Category | | | |
| Dependent Variable: BMI Category | | | | Dependent Variable: BMI Category | | | |
| 1=Underweight, 2 = Normal Weight, 3 = Overweight, 4 = Obese | | | | 1=Underweight, 2 = Normal Weight, 3 = Overweight, 4 = Obese | | | |
| Significance: * = 10%, ** = 15%, *** = 1% | | | | Significance: * = 10%, ** = 15%, *** = 1% | | | |
| ^E^= Estimated value from Stage 1 | | | | ^E^= Estimated value from Stage 1 | | | |

| NLSY97: HLGM Results by Gender | | | | | |
| --- | --- | --- | --- | --- | --- |
| Male | | | Female | | |
| Age 12-17 | | | | | |
| Value | BMI Category | Observations | Value | Smoking Category | Observations |
| 0 | Underweight | 28 | 0 | Underweight | 61 |
| 1 | Normal Weight | 501 | 1 | Normal Weight | 455 |
| 2 | Overweight | 175 | 2 | Overweight | 126 |
| 3 | Obese | 59 | 3 | Obese | 69 |
| Modeling the probability of having a lower BMI Category | | | | | |
| Fit Statistics | | | Fit Statistics | | |
| -2 Log Likelihood | 1101.41 |  | -2 Log Likelihood | 1127.06 |  |
| AIC | 1,127 |  | AIC | 1,153.06 |  |
| Results | | | Results | | |
| Effect | Estimate | Std Dev | Effect | Estimate | Std Dev |
| Intercept (Normal Weight) | -4.0378 | 2.6348 | Intercept (Normal Weight) | -5.7308 | 4.5183 |
| Intercept | 12.007*** | 2.7077 | Intercept | 9.9796** | 4.3622 |
| Intercept (Obese) | 19.4281*** | 2.8045 | Intercept (Obese) | 16.4264*** | 3.8818 |
| Smoking | -0.02014 | 0.02023 | Smoking | -0.03231 | 0.02226 |
| Age | -0.3747** | 0.1307 | Age | -0.2728 | 0.1966 |
| South | 1.6192** | 0.6405 | South | -0.02474 | 0.699 |
| Northeast | 0.2191 | 0.7926 | Northeast | 2.8537** | 0.9309 |
| Black | -0.9258 | 0.7729 | Black | -4.1076** | 1.2962 |
| Hispanic | -2.9809*** | 1.0109 | Hispanic | -5.9523** | 1.9681 |
| Urban | 1.8871*** | 0.6158 | Urban | 0.614 | 0.5952 |
| Household Size | 0.04371 | 0.1614 | Household Size | 0.0198 | 0.1765 |
| logIncome/Poverty | -0.00026 | 0.2194 | logIncome/Poverty | 0.4183* | 0.2392 |
| Age 20-25 | | | | | |
| Value | BMI Category | Observations | Value | Smoking Category | Observations |
| 0 | Underweight | 50 | 0 | Underweight | 122 |
| 1 | Normal Weight | 1,720 | 1 | Normal Weight | 1,577 |
| 2 | Overweight | 1,187 | 2 | Overweight | 649 |
| 3 | Obese | 605 | 3 | Obese | 519 |
| Modeling the probability of having a lower BMI Category | | | | | |
| Fit Statistics | | | Fit Statistics | | |
| -2 Log Likelihood | 5,212.34 |  | -2 Log Likelihood | 4,245.53 |  |
| AIC | 5,212.44 |  | AIC | 4,271.53 |  |
| Results | | | Results | | |
| Effect | Estimate | Std Dev | Effect | Estimate | Std Dev |
| Intercept (Normal Weight) | -0.7993 | 1.0955 | Intercept (Normal Weight) | 0.6381 | 1.2337 |
| Intercept | 9.8061*** | 1.1263 | Intercept | 15.3828*** | 1.596 |
| Intercept (Obese) | 15.7323*** | 1.1998 | Intercept (Obese) | 20.3435*** | 1.7886 |
| Smoking | 0.01879** | 0.008182 | Smoking | 0.01333 | 0.01056 |
| Age | -0.4389*** | 0.04221 | Age | -0.4276*** | 0.05117 |
| South | 0.004513 | 0.3257 | South | -0.1617 | 0.3802 |
| Northeast | -0.2792 | 0.4674 | Northeast | -0.2587 | 0.4919 |
| Black | -0.2936 | 0.5207 | Black | -6.9422*** | 1.1584 |
| Hispanic | -2.3266*** | 0.5183 | Hispanic | -1.0463 | 0.7553 |
| Urban | 0.6573** | 0.2051 | Urban | -0.1091 | 0.2205 |
| Household Size | 0.01598 | 0.05067 | Household Size | -0.1803** | 0.05937 |
| logIncome/Poverty | 0.004038 | 0.07045 | logIncome/Poverty | 0.1664** | 0.08663 |
| Age 27-32 | | | | | |
| Value | BMI Category | Observations | Value | Smoking Category | Observations |
| 0 | Underweight | 21 | 0 | Underweight | 67 |
| 1 | Normal Weight | 993 | 1 | Normal Weight | 973 |
| 2 | Overweight | 1,068 | 2 | Overweight | 548 |
| 3 | Obese | 638 | 3 | Obese | 594 |
| Modeling the probability of having a lower BMI Category | | | | | |
| Fit Statistics | | | Fit Statistics | | |
| -2 Log Likelihood | 3882.85 |  | -2 Log Likelihood | 3412.8 |  |
| AIC | 3,909 |  | AIC | 3,438.80 |  |
| Results | | | Results | | |
| Effect | Estimate | Std Dev | Effect | Estimate | Std Dev |
| Intercept (Normal Weight) | -8.9651*** | 2.2819 | Intercept (Normal Weight) | -2.2284 | 1.5557 |
| Intercept | 3.0692 | 2.0477 | Intercept | 8.7085*** | 1.6764 |
| Intercept (Obese) | 13.4159*** | 1.7317 | Intercept (Obese) | 13.0841*** | 1.7451 |
| Smoking | 0.01566** | 0.01157 | Smoking | -0.02052 | 0.01203 |
| Age | -0.3145*** | 0.05917 | Age | -0.2136*** | 0.04834 |
| South | 0.5757 | 0.4572 | South | -0.0625 | 0.4652 |
| Northeast | 1.8629** | 0.7693 | Northeast | 0.6585 | 0.5953 |
| Black | -0.568 | 0.7037 | Black | -4.8432*** | 0.9315 |
| Hispanic | -2.2747*** | 0.6956 | Hispanic | -1.8334** | 0.8562 |
| Urban | 0.1802 | 0.2885 | Urban | -0.1441 | 0.2868 |
| Household Size | 0.009861 | 0.07968 | Household Size | -0.1697** | 0.07814 |
| logIncome/Poverty | 0.2214* | 0.1134 | logIncome/Poverty | 0.04544 | 0.1091 |
| Dependent Variabl: BMI Category 1=Underweight, 2 = Normal Weight, 3 = Overweight, 4 = Obese | | | | | |
